# Usability and Credibility of a COVID-19 Vaccine Chatbot for Young Adults and Health Workers in the United States: Formative Mixed Methods Study

**DOI:** 10.2196/40533

**Published:** 2023-01-30

**Authors:** Rose Weeks, Pooja Sangha, Lyra Cooper, João Sedoc, Sydney White, Shai Gretz, Assaf Toledo, Dan Lahav, Anna-Maria Hartner, Nina M Martin, Jae Hyoung Lee, Noam Slonim, Naor Bar-Zeev

**Affiliations:** 1 International Vaccine Access Center Department of International Health Johns Hopkins Bloomberg School of Public Health Baltimore, MD United States; 2 Johns Hopkins Center for Indigenous Health Department of International Health Johns Hopkins Bloomberg School of Public Health Baltimore, MD United States; 3 Stern School of Business New York University New York, NY United States; 4 Whiting School of Engineering Johns Hopkins University Baltimore, MD United States; 5 IBM Research AI Haifa Israel; 6 Department of International Health Johns Hopkins Bloomberg School of Public Health Baltimore, MD United States

**Keywords:** COVID-19, chatbot development, risk communication, vaccine hesitancy, conversational agent, health information, chatbot, natural language processing, usability, user feedback

## Abstract

**Background:**

The COVID-19 pandemic raised novel challenges in communicating reliable, continually changing health information to a broad and sometimes skeptical public, particularly around COVID-19 vaccines, which, despite being comprehensively studied, were the subject of viral misinformation. Chatbots are a promising technology to reach and engage populations during the pandemic. To inform and communicate effectively with users, chatbots must be highly usable and credible.

**Objective:**

We sought to understand how young adults and health workers in the United States assessed the usability and credibility of a web-based chatbot called Vira, created by the Johns Hopkins Bloomberg School of Public Health and IBM Research using natural language processing technology. Using a mixed method approach, we sought to rapidly improve Vira’s user experience to support vaccine decision-making during the peak of the COVID-19 pandemic.

**Methods:**

We recruited racially and ethnically diverse young people and health workers, with both groups from urban areas of the United States. We used the validated Chatbot Usability Questionnaire to understand the tool’s navigation, precision, and persona. We also conducted 11 interviews with health workers and young people to understand the user experience, whether they perceived the chatbot as confidential and trustworthy, and how they would use the chatbot. We coded and categorized emerging themes to understand the determining factors for participants’ assessment of chatbot usability and credibility.

**Results:**

In all, 58 participants completed a web-based usability questionnaire and 11 completed in-depth interviews. Most questionnaire respondents said the chatbot was “easy to navigate” (51/58, 88%) and “very easy to use” (50/58, 86%), and many (45/58, 78%) said its responses were relevant. The mean Chatbot Usability Questionnaire score was 70.2 (SD 12.1) and scores ranged from 40.6 to 95.3. Interview participants felt the chatbot achieved high usability due to its strong functionality, performance, and perceived confidentiality and that the chatbot could attain high credibility with a redesign of its cartoonish visual persona. Young people said they would use the chatbot to discuss vaccination with hesitant friends or family members, whereas health workers used or anticipated using the chatbot to support community outreach, save time, and stay up to date.

**Conclusions:**

This formative study conducted during the pandemic’s peak provided user feedback for an iterative redesign of Vira. Using a mixed method approach provided multidimensional feedback, identifying how the chatbot worked well—being easy to use, answering questions appropriately, and using credible branding—while offering tangible steps to improve the product’s visual design. Future studies should evaluate how chatbots support personal health decision-making, particularly in the context of a public health emergency, and whether such outreach tools can reduce staff burnout. Randomized studies should also be conducted to measure how chatbots countering health misinformation affect user knowledge, attitudes, and behavior.

## Introduction

The internet’s continual availability, breadth of coverage, interactivity, and anonymity has made it a preferred health information source [1]; however, it has also propagated the spread of scientifically inaccurate, false, or misleading health information [2-4]. The COVID-19 pandemic has taken an enormous toll on human health and social functioning, raising novel and substantial challenges in communicating reliable and dynamically changing health information to a broad and sometimes skeptical public [5-9]. Although COVID-19 vaccines are thoroughly studied, misinformation abounds and is widely shared [10]. A survey in May 2021 of over 5 million Americans found adults aged 18-34 years had the highest rates of vaccine hesitancy [11], with this and other studies citing concerns regarding vaccine development, safety, and effectiveness [12-14]. This has hampered vaccine uptake in the United States, which experienced extraordinarily high COVID-19 mortality relative to other high-income countries [15].

We sought to provide access to reliable, relevant, and up-to-date information through the development of an automated dialog system, or chatbot, which supported direct questioning and engagement by users on their own terms and in their own words. Chatbots were seen early in the pandemic as a promising technology to reach and engage populations [16,17]. Chatbot performance has improved enormously in recent years, and they provide individuals with support on diverse health issues from depression to weight management [18,19]. Mental health chatbots have been shown to improve self-reported measures of depression [20]. Very limited evidence points to the potential health impact of providing vaccine information through chatbots. Experiments with crowd workers indicate that time spent engaging with a chatbot may be related to improved outcomes such as attitudinal changes related to vaccine acceptance, a promising finding that argues for making chatbot platforms compelling and engaging to incentivize chatting long enough for the intervention exposure to be sufficiently meaningful [21,22]. Chatbots must be seen by their intended users as highly usable and credible*.* Usability describes the effectiveness, efficiency, and satisfaction with which targeted users complete tasks on a tool in a specific context [23,24]. Credibility reflects a combination of integrity, dependability, and competence [25,26]; users judge a website’s credibility by assessing its origins, content, context, functionality, and design [27,28].

At the cusp of COVID-19 vaccine authorization for US adults aged over 18 years, we developed a web-based chatbot with an illustration of a smiley emoji in warm orange and yellow tones, embodying a friendly bot presenting credible facts about COVID-19 vaccines [29-31]. The chatbot, called Vira, short for Vaccine Information Resource Assistant, was available on a website, accessible on WhatsApp, and embedded in several other websites, such as city health departments, via an embed code snippet. IBM Research developed and managed the chatbot’s backend, which is based on a neural model that maps each user utterance to (at most) one concern from a predefined list of concerns, referred to as *Key Points*. Key Points were identified through various means: using a Twitter analysis, reviewing audience questions in Zoom-based public forums hosted by authors’ affiliated academic centers, and synthesizing web pages with frequently asked questions [32-34]. In addition to surfacing emerging concerns from the logs, the backend used Key Point Analysis, a commercially available technology that facilitates extractive summarization to process numerous comments, opinions, and statements and reveal the most significant points and their relative prevalence [35,36]. Vira was initially trained to respond to 100 Key Points with up to 4 alternative responses per concern to minimize repetition and enhance the naturalness of Vira’s dialog [37].

To investigate Vira’s reception with our targeted users, we sought to understand their COVID-19–related concerns, experience using other chatbots, and preferences related to information seeking. Qualitative evidence describing the experiences of health consumers, particularly racial and ethnic minority women, with health chatbots and other digital health tools is limited [38-41]. Understanding people’s needs and preferences, as told in their own words, was critical to developing a human-centered platform deemed by intended users as effective and appropriate. This study, therefore, sought to (1) understand how users assessed the chatbot’s usability and credibility and describe their intention to use the chatbot and (2) apply this understanding to improve the user experience.

## Methods

### Recruitment

We recruited two participant groups in urban US communities: (1) individuals aged 18-28 years; and (2) health workers, who were individuals contracted or employed by health departments to encourage the uptake of COVID-19 vaccines. We posted ads on Craigslist and Twitter targeting young people and health workers in Baltimore, Charlotte, New York City, Philadelphia, and Washington, D.C., and we used snowball sampling through professional contacts to identify health workers. We sought to achieve variability along lines of race and ethnicity to represent our targeted users. For both groups, we excluded people who stated they would “definitely NOT choose to get a COVID-19 vaccine by August 2021” in a scaled response, since the chatbot aimed to target users along the vaccine hesitancy continuum excepting those refusing vaccines [42-44].

Users were invited to participate in 3 possible activities—a web-based questionnaire, Zoom-based interview, or a web-based focus group discussion—described elsewhere [30]. Participants were given US $20 Amazon e-gift cards for each study activity completed.

### Data Collection and Analysis

#### Usability Questionnaire

To understand Vira’s overall acceptability and its ability to respond appropriately, we presented the website and chat function to target users. We invited users to complete a Qualtrics-based written consent form, followed by a web-based Qualtrics-based questionnaire, with both forms in English. The questionnaire asked participants 10 open-ended and scaled questions about COVID-19 vaccine beliefs, previous chatbot experiences, the potential use of a chatbot to seek information about COVID-19 vaccines, and anticipated barriers. The Chatbot Usability Questionnaire (CUQ) was also included, which is a validated instrument that asked 16 questions about the chatbot’s persona, chat initiation, navigation, precision, responses, and error handling rated on a scale of 1 (strongly disagree) to 5 (strongly agree) [45,46]. We summarized the survey and CUQ responses using descriptive statistics. We also analyzed themes from the open-ended questions through cross-case comparisons, grouping responses for each question and assessing similarities and differences across responses.

Although no direct comparison can be made to other chatbot assessments, we sought to make our usability assessment results understandable to those familiar with the System Usability Scale. Therefore, we calculated participant responses to the CUQ out of 64 using the formula in equation 1, then normalized to 100 [45]. Descriptive statistics of CUQ scores are presented in the results.

The CUQ calculation is as follows:



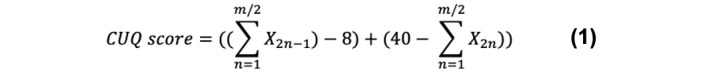



where *X_n_* is the score given by the participant on the *n*th question and m=6.

#### In-depth Interviews

**Figure 1:**
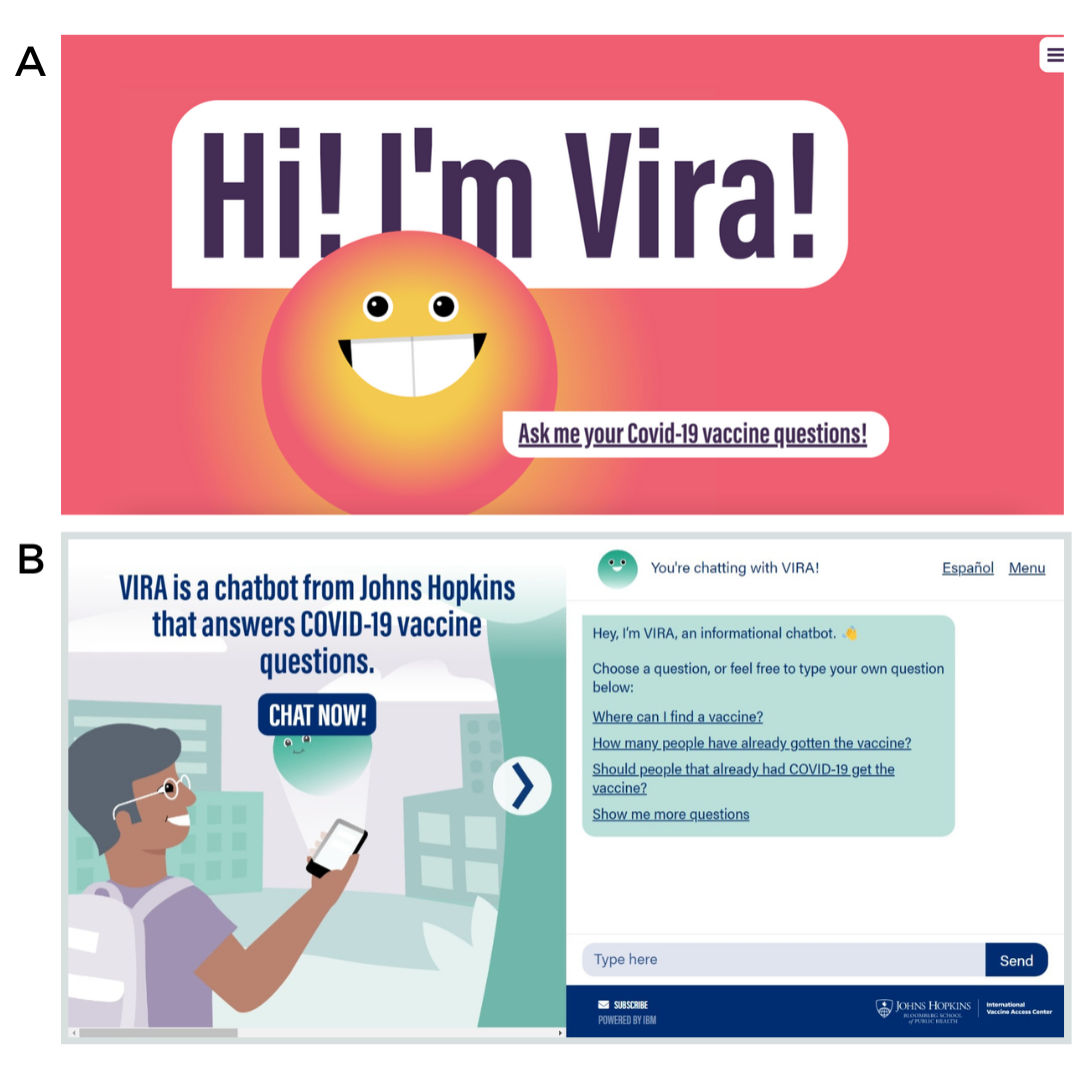
(A) Interface shown to participants. Study participants reviewed this user interface. (B) User interface, following study. The new interface at VaxChat.org incorporates feedback from study participants.

To solicit qualitative feedback on the chatbot’s usability, credibility, and users’ intention to engage with the tool, we conducted in-depth interviews (IDIs) with health workers and young people. Interviews were conducted in 2021 from June to October via Zoom videoconferencing software (licensed account; Zoom Video Communications Inc). After obtaining verbal consent from participants, we conducted 60-minute, audio-recorded interviews, exploring if users had difficulty using the chatbot and if they could identify intended audiences and use cases for the tool. Interviewers also asked whether the chatbot seemed trustworthy and confidential and probed to understand how users reacted to the bot persona, meaning the personality the bot assumes while interacting with a user [47]. See Figure 1A for a screenshot of the website the participants reviewed.

Recorded interviews were transcribed using Temi transcription software (Temi) and uploaded to Dedoose (version 9.0.46; SocioCultural Research Consultants, LLC), a web-based qualitative data management software. A thematic codebook was developed using a deductive grounded theory approach. First, one team member created a codebook derived from the semistructured IDI guide, with team members involved in facilitating IDIs collectively updating the codebook. Then, 2 members piloted the codebook with a handful of transcripts, noting missing codes as well as coding discrepancies. Once the codebook was finalized, 2 members coded each transcript, and a third reviewed coded text segments for discordant coding. Throughout this process, memos were used to organize and document the analytic process.

During reassembly, we grouped textual excerpts related to codes, scanning segments to discover conceptual concurrence and discord between participants and participant types (eg, opinions shared by young people but not health workers). We also identified textual data reinforcing quantitative and qualitative findings from the usability questionnaire, specifically around themes of usability and credibility.

### Ethics Approval

This formative study was approved by the Johns Hopkins Bloomberg School of Public Health Institutional Review Board (protocol number 15714).

## Results

### Participant Characteristics 

#### Usability Questionnaire Characteristics

As shown in Table 1, 58 participants completed the usability questionnaire, among whom 40 (69%) were female, with 42 (72%) holding a bachelor’s degree or higher, 10 (17%) having some college or an associate degree, and the remaining 6 (10%) having a high school diploma. In all, 3 (5%) participants reported being unvaccinated against COVID-19.

**Table 1:** Participants’ self-reported demographic characteristics.

Characteristic		IDI^a^ (n=11), n (%)	Usability questionnaire (n=58), n (%)
**Gender (IDI: n=11; usability questionnaire: n=58)**			
	Female	9 (82)	40 (69)
	Male	1 (9)	16 (28)
	Nonbinary	1 (9)	2 (3)
**Education (IDI: n=11; usability questionnaire: n=58)**			
	Bachelor’s degree or higher	11 (100)	42 (72)
	Associate degree	0 (0)	6 (10)
	Some college, no degree	0 (0)	4 (7)
	High school graduate	0 (0)	6 (10)
**Race or ethnicity (IDI: n=11; usability questionnaire: n=57)**			
	American Indian and Alaska Native	1 (9)	1 (2)
	Asian	1 (9)	8 (14)
	Black or African American	2 (18)	16 (28)
	Hispanic	0 (0)	5 (9)
	White	7 (64)	27 (47)
**Age (years; IDI: n=11; usability questionnaire: n=58)**			
	8-24	3 (27)	30 (52)
	25-49	7 (64)	28 (48)
	50-69	1 (9)	0 (0)
**Income (US $; IDI: n=10; usability questionnaire: n=51)**			
	<40,000	5 (50)	24 (47)
	40,001-60,000	3 (30)	10 (20)
	60,001-80,000	1 (10)	7 (14)
	80,001-100,000	1 (10)	9 (17)
	>100,000	0 (0)	1 (2)
**COVID-19 vaccination status (IDI: n=11; usability questionnaire: n=58)**			
	Vaccinated	11 (100)	55 (95)
	Unvaccinated	0 (0)	3 (5)

^a^IDI: in-depth interview.

#### Interview Participant Characteristics

Out of 11 total participants, 9 (81%) were aged 18-28 years, including 4 (36%) who worked as health workers; 2 (18%) IDI participants were health workers aged >28 years; all but 2 participants identified as female (9/11, 81%); and all were previously vaccinated against COVID-19. Of these participants, 6 (55%) also completed the web-based questionnaire.

### Quantitative Results: CUQ

We assessed the functionality or ease of navigation with the CUQ. Questionnaire results, displayed in Table 2, indicate that most participants agreed with the statement that the chatbot was “easy to navigate” (51/58, 88%) and “easy to use” (50/58, 86%), with a corresponding proportion disagreeing that it was “very complex” (47/58, 81%). Half (29/58, 50%) of the questionnaire respondents agreed that the chatbot “understood me well,” and 74% (42/57) disagreed that it would be “easy to get confused when using the chatbot.” Additionally, 91% (53/58) of respondents disagreed that “the chatbot seemed unfriendly” but only half (32/58, 55%) felt the personality was realistic and engaging. Finally, 43% (25/58) disagreed with the statement that “the chatbot seemed too robotic.”

CUQ scores, normalized out of 100, were calculated for 56 of the 58 participants; 2 participants did not complete all 16 questions within the questionnaire. The mean CUQ score was 70.2 (SD 12.1) with a median score of 70.3 (range 40.6-95.3).

**Table 2:** Chatbot Usability Questionnaire Results.

Scale items			Respondents, n/N (%)
**Positive scale items (Strongly Agree OR Agree)**			
	The chatbot was easy to navigate	51/58 (88)	
	The chatbot was easy to use	50/58 (86)	
	The chatbot was welcoming during initial setup	45/58 (78)	
	Chatbot responses were useful, appropriate, and informative	40/58 (70)	
	That chatbot explained its scope and purpose well	37/58 (64)	
	The chatbot’s personality was realistic and engaging	32/58 (55)	
	The chatbot understood me well	29/58 (50)	
	The chatbot coped well with any error or mistakes	28/57 (49)	
**Negative scale items (Strongly Disagree OR Disagree)**			
	The chatbot seemed unfriendly	53/58 (91)	
	The chatbot was very complex	47/58 (81)	
	The chatbot gave no indication as to its purpose	46/58 (79)	
	Chatbot responses were irrelevant	45/58 (78)	
	It would be easy to get confused when using the chatbot	42/57 (74)	
	The chatbot was unable to handle any errors	39/58 (67)	
	The chatbot failed to recognize a lot of my inputs	36/58 (62)	
	The chatbot seemed too robotic	25/58 (43)	

### Qualitative Results: Chatbot Usability

**Figure 2:**
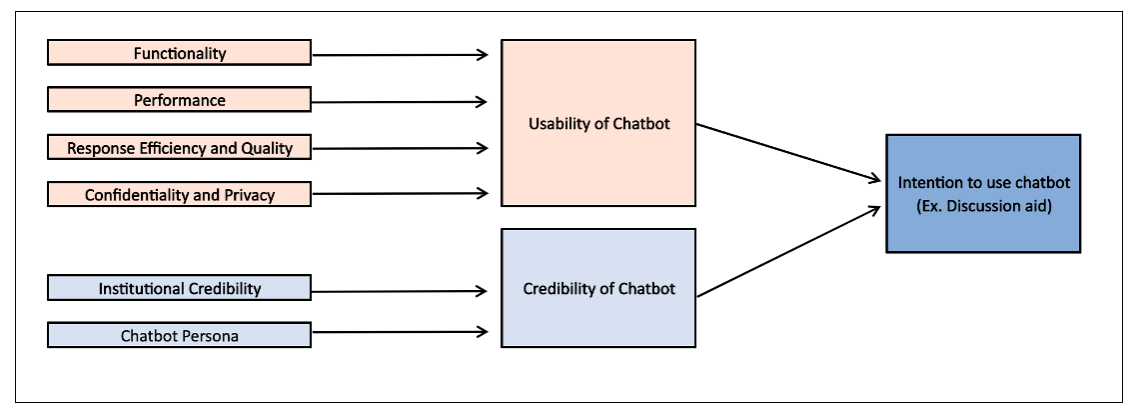
Conceptual schema derived from interview participant responses.

Interview participants described four contributing factors necessary to achieve usability, as shown in Figure 2: (1) functionality, or ease of use/navigation; (2) performance, or the chatbot’s ability to understand and accurately respond to queries; (3) response efficiency and quality; and (4) confidentiality and privacy of the tool. Participants described two primary contributing factors to achieving credibility: (1) institutional credibility and (2) chatbot persona.

#### Functionality

IDIs explored Vira’s website design and usability. Regarding functionality, most participants said the chatbot was easy to use. A young woman (IDI03) said, “it’s pretty simple–you can just click on the questions that pop up and see what some basic facts are.” A minor issue noted by health workers was not seeing how to initiate a chat, such as a clear button-style indication of where to click (see Figure 1A).

#### Performance

IDIs also assessed how precisely the chatbot responded. Both young people and health workers discussed Vira’s responsiveness, commenting that they could enter questions in their own words and receive varied, appropriate responses. One young health worker (IDI01) felt that the chatbot’s ability to understand “full questions” made it “very user friendly” and more human-like:

She was able to comprehend like my human questions which makes her calm, complex. Cause I think she's more like a person.

However, Vira’s lack of personalization was noted as a barrier by health workers, who suggested the chatbot could segment users before responding; for instance, the chatbot could identify a “base level of knowledge,” a young health worker (IDI13) said. Health workers thought the responses should be more detailed, for instance, providing specifics related to clinical trials or providing a second-tier response expanding on a first answer.

#### Response Efficiency and Quality

Young interview participants described multitasking and avoiding phone calls as key incentives for using Vira (see Table 3). Young people and one health worker compared the chatbot favorably to receiving advice from a medical professional, saying they felt the chatbot may be less biased and better informed on pandemic guidance and was available for free. A young participant (IDI06) said they felt less pressure with Vira to “watch my words” compared to when they talked to their doctor because their views on vaccines may not align with their doctor’s opinions.

**Table 3:** Enabling and hindering factors for a COVID-19 chatbot. Summary of in-depth interview (IDI) and open-ended questionnaire responses regarding anticipated benefits and barriers of chatbot use, nonspecific to the Vira chatbot.

Question, theme			Explanatory quotes (quote from questionnaire or in-depth interview with participant gender, ID number, and health worker status, if applicable)
**What would motivate you to use a COVID-19 vaccine chatbot?**			
	Efficiency	“condensed place for information,” gets “straight to the point” with a “one-tap process.” (young woman, IDI07; young female health worker, IDI01; and young female health worker, IDI08)	
	Avoid human interaction	“people my age...don’t want to make phone calls...just stay with the internet without actually having to communicate with a real person.” (young female health worker, IDI10)	
	Confidentiality and lack of judgment	“getting information without judgment” (young woman, P54) “For younger...16-20 year olds who feel they can’t talk to anyone...chatboxes are a great tool.” (young woman, IDI07)	
	Sharable tool to persuade others to get vaccinated	“test uncertainty that I hear from my friends to get an objective perspective” (young man, P41) “help spread positive information about the vaccine” (young woman, P64)	
**Do you foresee any barriers to using a chatbot?**			
	The expectation of rigid algorithmic design	“You’re kind of stuck in that rigid form of traveling through whatever path they’ve created through those algorithms.” (young female health worker, IDI13)	
	Query misunderstanding	“Frustration in getting the chatbot to understand what I want to learn or need to understand.” (young woman, P85)	
	Generic or nonpersonalized responses	“I would want to ask personalized questions about my own health” (young woman, P61)	
	No human interaction	“I really only found [chatbots] useful when I was able to communicate with a live person.” (young woman, P75)	
	Poor design	“I could see users getting frustrated quickly if...the bot is hidden somewhere on the webpage” (young woman, P74) “User interface design needs to be attractive with human [touch], images.” (female health worker, P105)	
	Concerns about accessibility for older generations	“older generations may find it difficult” (young woman, P75)	

#### Confidentiality and Privacy

Most young and health worker participants said they would use the chatbot to ask about sensitive issues and keep personal data out of a more commercial digital space. Several participants called the chatbot a “safe space” to ask questions. A young woman described this concept:

A lot of young people have family members who are anti-vaxxers. Having a chat box [sic] where they didn’t have to talk to an adult who might want to know how old they are, where their parents are, [would be] a safe space, for lack of better words.

One health worker (IDI04) who had used the Vira chatbot in her work described how she positioned it in conversations with community members:

I gave them the site while they were with me and told them to go home and ask some of the questions that they thought maybe were like dumb or didn’t want to like tell me, and they felt comfortable with doing that...that really helped in that instance.

Most participants, largely aged <30 years, were aware but not particularly worried about data privacy in this environment. A young man (IDI05) said when it came to data privacy, the chatbot compared favorably with web search engines, which “are going to take that information and use it for third-party information and ads...[the chatbot] is more private than public.” A young female health worker (IDI09) said:

...some people may feel like Hopkins is trying to gather data on what people are asking, and it very well might be, and that can still be confidential and private...I think that it would keep my information private for the most part.

### Qualitative Results: Credibility of Chatbot

Interview participants listed institutional credibility and chatbot persona as key factors contributing to their determination of the chatbot’s credibility. Their assessment encompassed judgments around the chatbot’s origin, how reliable the content appeared, how they felt about using it at this stage of the pandemic, and how website design influenced their decision to use the chatbot.

#### Institutional Credibility

Nearly all participants said the chatbot was trustworthy because it came from Johns Hopkins University, rather than from government or pharmaceutical sources. Participants noted that while the home page had a logo at the footer, the website was overall not clearly branded as a Johns Hopkins resource, which half of all participants noted as a missed opportunity to identify the resource as trustworthy. However, one young health worker (IDI08) said she did not think it was trustworthy because “we don’t know who’s sponsoring it...[and] where my information is going.”

#### Chatbot Persona

The chatbot’s “mascot,” as participants referred to the smiley emoji on the home page, elicited mixed responses. Young participants, including health workers aged <30 years, said it contrasted with the topic of COVID-19—sometimes favorably, other times poorly. The mascot was said to be “silly,” “goofy,” and “very happy,” which one young participant (IDI06) felt signaled that “it’s going to be a friendly chatbot.” A male participant (IDI05) agreed, saying “it’s not going to be like the news and media where it’s...doom and gloom.” However, even this participant felt the emoji mascot was “a little much,” and another young participant felt the mascot was “a little bit creepy.” A few young people compared the mascot to a Pokémon cartoon creature. Although 2 health workers did not express concern about the mascot’s credibility, half of all young participants said the design was inappropriately childish for a tool targeted toward users like them. Although many participants liked the “warm and inviting” colors, several described the website as pink, which one female participant said would be too “girly” for male users. Several participants noted the “bright” colors could be less “overpowering,” with 2 health workers suggesting the website should use lighter, cooler colors.

Several participants expressed uncertainty about the name “Vira.” Some were not sure how to pronounce it, and others said it conveyed an association with the coronavirus. When portrayed as an acronym—Vaccine Information Resource Assistant—VIRA was better understood and accepted.

Despite these issues and Vira’s somewhat “robotic” persona, as seen by persona-related usability scores in Table 2, three interview participants said that the chatbot would help allay their pandemic-related anxiety. Regarding a breakthrough COVID-19 case, a young woman (IDI03) said:

I would be very anxious and turn to something like this to find new information. It would help me calm down.

### Qualitative Results: Intention to Use

Young people said the tool would help in discussions with vaccine-hesitant friends, family members, or members of their community. In all, 9 (16%) out of 58 questionnaire respondents described using the chatbot to encourage others to get vaccinated (see exemplar quotes in Table 3).

The 6 interview participants who served as health workers felt the chatbot could support their work. First, they must keep on top of emerging concerns in the community and look up new questions. Second, listening one on one to individuals was an important part of their role. As one middle-aged health worker (IDI02) said,

We just give them a support system. They feel someone is hearing them, their issues, their opinions. They want to record their information. They want to make sure that someone is listening...[and] giving them value.

Participants described addressing the public’s concerns about vaccines via phone, for instance, in contact tracing or at health fairs, with many queries collapsing into a batch of common questions. As one young female health worker (IDI09) said:

I’ve gotten really backlogged with the amount of people that have called. There’s a lot of very similar questions. Some of them can be answered by a chatbot and it would probably streamline that process.

Health workers noted the potential of a chatbot as a source to easily access up-to-date content. As one young health worker (IDI01) said, once the resource was approved by her department of health, “I’d be using it like every time I don’t know an answer or honestly...just to double-check my work.”

## Discussion

### Principal Findings

This study took a mixed methods approach to measure the perceived usability and credibility of a COVID-19 vaccine information chatbot with natural language processing capability in a web-based chat environment. An ethnically and racially diverse sample of urban-dwelling young people and health workers assessed the chatbot as achieving high usability in that it was easy to use, performed well in understanding their inputs, and offered advantages over human interactions through efficiency, confidentiality, and reliability; noted usability deficits included the chatbot’s inability to personalize responses. In the domain of credibility, participants noted the institutional affiliation with Johns Hopkins as an asset and Vira’s inappropriately cartoonish visual persona as being an area for improvement. Young people and health workers, most of whom were already vaccinated, envisioned using the chatbot as a discussion aid to encourage others to seek out vaccines. Finally, interview participants offered clear guidance to comprehensively redesign Vira’s visual persona.

Vira’s usability scores compare favorably to those of other health chatbots evaluated through standardized measures. One study examining a health chatbot with majority White adults found a mean score of 61.6 out of 100, whereas an HPV vaccine counseling chatbot used by 24 mostly White young adults scored between 74 and 80 out of 100 [19,48]. Vira was very easy to use; provided useful, appropriate, and informative responses; and explained its purpose. Although only half of the respondents thought Vira understood well and coped with input errors, this is double the score seen in evaluations of other health chatbots, showing users’ expectations for response accuracy are high [49]. In interviews, participants appreciated that the chatbot—which can handle typos and shorthand (eg, “vax” for vaccine)—could understand full sentences, and they perceived a social-like encounter. In other chatbots, so-called social bonding increases user acceptability and confidence, influences persuasiveness, and alleviates anxiety [47,50]. Since users felt less self-conscious of how they phrased a question, they may have been freer to ask sensitive questions—or encourage others to do so from the privacy of their screens. The natural typing style gave the chatbot a human-like status, and the exchange became like a social interaction where there would be no real-world consequences for a perceived stupid or inappropriate question.

Users in our study noted that the chatbot was not personalized for them and did not customize responses regarding their baseline knowledge, attitudes, vaccination status, or individual health status (eg, underlying conditions). Many other health chatbots provide personalized content and conversations to improve user engagement, dialog quality, and self-reflection [51]. This is common across downloadable apps; a review of 78 health apps with chatbots noted that 60% of these included personalization features, with most (90%) apps personalizing content—some simply addressing users by name [18]. Vira’s design as a web-based app limited such functionality. However, participants cognizant of social discord around vaccination recognized the potential of the confidentiality and privacy offered by the anonymous web-based chatbot platform. In that the main challenge regarding personalization is privacy [37], this anonymity and the “safe space” offered by Vira may be weighed against personalization.

In terms of its credibility, Vira was rated as very friendly, with participants describing the tool as having the potential—with design iterations—to be a trustworthy source for information on COVID-19 vaccines, a politicized and emotive issue. Evidence supports the notion that chatbots presenting information about controversial topics can be convincing and trustworthy, especially with supporting links [52,53]. Many users of health chatbots report high satisfaction and positive perceptions, and the use of even moderately rated chatbots has been associated with behavior changes [19,20]. Although this study was not designed to evaluate the effectiveness of Vira in changing participant attitudes, their appraisal of the chatbot as being highly usable supports potential pathways toward behavior change to explore further.

Participants stated an interest in using Vira in their personal lives and, in the case of health workers, in professional roles. Large health organizations evidently understand the potential of chatbot technology, with the health care chatbot market expected to reach nearly $US 1 billion by 2027 [54]. Health agencies and some US states have also launched health chatbots during the COVID-19 pandemic to encourage widespread sharing of credible health information [55-58]. Further investments in high-performing, user-validated chatbots would aid health educators in communicating about rapidly changing vaccination guidance. In addition, installing chatbots on health department websites could reduce call volume and support public health workers [59].

This formative study provided investigators with user feedback to iteratively improve the user experience for Vira, a chatbot designed rapidly to support vaccine queries during the pandemic’s peak, including on the visual persona, provided rapid feedback for a website redesign (see Figure 1B). The new VIRA, spelled in all capital letters, is shown in calming blue and purple tones as a smaller, still-friendly orb supporting human users. The chatbot’s response database, or repository of potential responses, was also comprehensively edited via a separately reported message testing study [30].

However, numerous questions remain: What are the social implications of automating conversations about vaccine decisions, previously a person-to-person encounter highly reliant on trust? As mental health chatbots can reduce anxiety and loneliness, can vaccine chatbots simulate a support system validating people’s search for answers—helping them feel heard, even if by a bot? Evidence is needed, through a randomized evaluation, to explore which elements of a chatbot interaction, such as chat duration or added personalization, could lead to measurable changes in vaccine attitudes and behavior or, indeed, impact related to other stigmatized health issues such as sexual health. Whether chatbots effectively counter health misinformation and support the use of motivational interviewing, one of the only evidence-based means to soften vaccine hesitancy, is another important area for exploration [60,61].

Although this study provided rapid feedback to course-correct Vira’s visual design and inform its outreach strategy, it has several limitations. First, our participants were mostly college-educated, perhaps due to a reliance on Twitter ad recruitment [62]. In addition, participants were recruited using Johns Hopkins–branded ads and may have been more favorable toward the institution than others who did not click on the ads. IDI recruitment by Johns Hopkins of several health workers employed in Baltimore likely introduced bias, since participants may have been less likely to offer unfavorable comments out of a sense of collegiality; nonetheless, such participants did offer specific critiques. The conduct of the study at a university widely known to promote COVID-19 vaccination likely dissuaded some vaccine skeptics [63]. Since investigators needed to collect data rapidly to alter a tool already in use during a pandemic, we conducted a small number of qualitative interviews, focusing on professional users. Nearly all our participant sample said they had been vaccinated, which is not representative of young adults and health workers in the United States; however, a large proportion of the US public who got primary doses of COVID-19 vaccines have not subsequently obtained booster doses [64]. Therefore, we believe the results are relevant to support efforts to counteract vaccine hesitancy. Further, most participants describe theoretical usefulness in a one-time encounter with the chatbot. A final limitation is that this study describes Vira’s performance at launch; since then, the number of Key Points Vira can address has nearly doubled, and the performance of its adaptive algorithm has presumably improved.

### Conclusions

Launched at the peak of the COVID-19 pandemic, as cases caused by the Delta variant crested in the US, Vira offered a highly functional and credible system to respond quickly and appropriately to users’ vaccine queries. We used rich data gathered through interviews to identify and remedy deficits in the chatbot persona. Young people and health workers in the study felt chatbots offered significant benefits in a pandemic context due to their reliability, responsiveness, and efficiency and that the Vira chatbot was a credible and private way to seek information on a sensitive issue. More research is needed to determine how guidance offered in an anonymous 2-way dialog, potentially designed to simulate a motivational interview, could shift perceptions of emotionally charged issues with participants in a real-world setting. Evidence is also needed to measure whether chatbots strengthen public education services and are cost-effective if made widely available.

## Abbreviations

CUQ: Chatbot Usability Questionnaire
IDI: in-depth interview
Vira: Vaccine Information Resource Assistant
